# Disinfection after the Occurrence of Infectious Diseases

**Published:** 1898-04

**Authors:** S. W. Abbott

**Affiliations:** Boston, Mass.


### DISINFECTION AFTER THE OCCURRENCE OF INFECTIOUS DISEASES.

By S. W. ABBOTT, M. D., Boston, Mass.

It is not my intention in this brief paper to present an exhaustive statement of the subject of disinfection, but to present a brief digest for practical use.

The object of disinfection is the destruction of infection, or infectious material (in the words of one of the best authors, the *contagium vivum* of disease). The practical question then, in each case of infectious diseases, is *what is the best disinfectant for use in this particular case, and how shall it be applied?*

Infectious diseases differ in their character, their virulence, and their mode of transmission, as much as do the various diseases of plants, and the different insect pests with which the agriculturist has to deal, and for each of which he must provide himself with some particular insecticide, as a weapon of defense. Now it is Paris green, now tobacco smoke, now whale-oil soap, etc.

Just so, different disinfectants are needed for different purposes, and as medicine is a progressive science, so the science of disinfection to-day is quite different from that of a dozen years ago.

Let us review very briefly, the principal means of disinfection in use at the present time, and the methods of their use.

First. Actual *destruction by fire*. This is undoubtedly the most thorough and certain method, but is only applicable to organic substances and such as are worthless, or can not be disinfected by other methods. It may be applied to infected clothing, rags and bits of infected cloth, such as are contaminated with infected discharges from the mouth, nose, throat and other parts of the body. Mattresses, saturated with infectious material, upholstery and cheap furniturne, which are not deemed worth disinfecting by other methods, may thus be treated. Infected school books and library books, especially when worn and of little value, may be destroyed by fire, in preference to running the risk of using them without treatment.*

In the treatment of one or more cases of small-pox, in the milder seasons of the year, the writer has found an efficient method to be the selection of a suitable location, remote from the inhabited districts, and there treating the patient in a good army tent, which can be burned after the recovery or death of the patient, with the bed, bedding and furniture.

Second. The *application of heat*.

(a.) *Dry heat*. Exposure to a heat of 150° to 250° F., according to the nature of the disease for which disinfection is

---

*Formalin has been recommended for disinfecting books, but its use for this purpose does not yet appear to be settled. Authorities differ with reference to the practicability of disinfecting the interior of a tightly closed book.

required. Special apparatus is required, and the method is inconvenient for ordinary use, in consequence of the liability to overheating and consequent injury of the fabrics, or substances to be disinfected. (This statement does not apply to the use of sterilizers in hospitals and public institutions having the temperature under careful management and control.)

(b.) *Moist heat.* (1.) *Steam.* Steam disinfectors are now in use at all the principal quarantine stations, and in the large cities for the disinfection of infected clothing, bedding, mattresses, carpets, metallic surgical instruments, and all material which is not liable to injury by steam. Every city of 25,000 inhabitants or more ought to have at least one such steam disinfector, either portable or stationary, for use under the direction of the local board of health. (Good descriptions of such apparatus may be found in the reports of the boards of health of large cities—not only of Paris and Berlin, but also of New York, Philadelphia and Boston.) (2.) *Boiling water.* This may be used for bed linen, clothing, sputum cups, cuspidors and metallic surgical instruments, metal and crockery ware.

Third. *Chemical disinfectants* have well-defined powers for the destruction of disease germs, as determined by experiment. Of these, the most efficient and useful in liquid form, are solutions of carbolic acid, chloride of lime, bichloride of mercury, lime water and milk-of-lime. A good disinfectant for general use should be inexpensive, readily soluble in water, and capable of easy and rapid application. As a general rule, disinfectants in the crude form and in bulk, are both cheaper and more efficient than patented articles.

*Crude carbolic acid* is soluble in water in the ratio of about one part to 30. (About four ounces to the gallon.) A solution of one part in 400 will destroy most bacilli, but not their spores.

*Bichloride of mercury.* (Corrosive sublimate.) One part dissolved in 1000 parts of water will suffice to destroy disease germs of all sorts in less than ten minues, and ordinary bacilli,* in less than one minute. The extremely poisonous character of this solution makes it necessary to take special precautions in its use. Its property of forming insoluble compounds with albumen, also seriously interferes with its efficiency.

*Chloride of lime.* This is one of the most efficient disinfectants and may be used either in powder or in solution; in the latter case, in the proportions of two parts of chloride of lime to 100 of water.

Either of the foregoing may be employed for application to walls, floors or wood-work.

*Milk of lime.* (Common whitewash.) This constitutes a very

**Ordinary bacilli*, i. e., most common forms of infectious diseases. The spores of anthrax require longer time, but anthrax is a disease of rare occurrence in the U. S., and when it does occur as a result of infection from the lower animals, it rarely spreads.

efficient disinfectant for use in the disinfection of excreta, by mixing it thoroughly with the excreta in equal bulk. It may be prepared by sprinkling a quart of water gradually over a quart of quick-lime in broken pieces in a wooden or metallic vessel. When the lime is reduced to powder, three quarts or more of water should be added, and the mixture allowed to stand. This was highly recommended by the German government in its cholera circular of 1892. It may be used for the flooding of areas and concrete floors in liberal quantities.

Weaker disinfectants are the permanganate of potash, and the sulphates of iron, copper and zinc. Either of which may be employed in a 5 per cent. solution.

*Gaseous disinfectants.* The principal disinfectants in gaseous form are sulphurous acid, chlorine and formaldehyde. Disinfectants of this character are employed for the disinfection of rooms which have been occupied by the sick. Chlorine gas is but little used in consequence of its extremely irritating nature. The use of sulphur is also rapidly losing ground in consequence of its foul odor, the occasional injury caused by it, and the fact that it frequently fails to accomplish its end, as shown by experiment. When used, the sulphur should be broken up and burned in an iron vessel, in such a manner as not to endanger the safely of the wood-work, since fire is not an uncommon occurrence, if it is carelessly used. At least three pounds shonld be used for each 1000 feet of air-space, and the disinfection will be much more thorough in the presence of moisture.

Recently the use of *formaldehyde* has come to the front very prominently as a gaseous disinfectant for use in apartments after infectious diseases. Its ready application makes it quite desirable for this purpose, and the best authorities, as quoted in the late numbers of Koch's *Zeitschrift*, show that it can be depended upon for the disinfection of surfaces, but not where penetrating power is required. In this direction, too much can not be expected of any disinfectant. For example, nothing has yet been found which will thoroughly disinfect the interior of a tightly-packed bale of rags, in a reasonable length of time. It is also difficult to disinfect the interior of closed school books or library books, provided the leaves are not loosely opened out. I mention this as a practical matter, since books are often exposed to infection, when used by sick children.

Every city and town should be provided with some good form of apparatus for the generation of formaldehyde, for disinfecting purposes.—*Richmond Journal of Practice.*

